# The *Photorhabdus* Virulence Cassettes RRSP-Like Effector Interacts With Cyclin-Dependent Kinase 1 and Causes Mitotic Defects in Mammalian Cells

**DOI:** 10.3389/fmicb.2020.00366

**Published:** 2020-03-13

**Authors:** Xia Wang, Jiawei Shen, Feng Jiang, Qi Jin

**Affiliations:** NHC Key Laboratory of Systems Biology of Pathogens, Institute of Pathogen Biology, Chinese Academy of Medical Sciences and Peking Union Medical College, Beijing, China

**Keywords:** *Photorhabdus asymbiotica*, PVC, effector, RRSP, cell mitosis

## Abstract

The “*Photorhabdus* virulence cassettes” (PVCs) secreted by *Photorhabdus* are defined as “extracellular contractile injection systems” (eCISs) and can deliver effectors to eukaryotic hosts for cytotoxicity. Previously, we demonstrated the cryogenic electron microscopy (cryo-EM) structure and assembly process of an intact PVC particle from *Photorhabdus asymbiotica*. In this work, we characterized the biological functions of a PVC effector, which is defined as a homologous protein of Ras/Rap1-specific endopeptidase domain (RRSP) in the multifunctional autoprocessing repeats-in-toxin (MARTX) toxin from *Vibrio vulnificus*. In this work, we found that the RRSP homologous protein (RRSP_Pa_) was associated with inhibition of cell proliferation and increased cell apoptosis and death of HeLa cells. Furthermore, we discovered that RRSP_Pa_ disturbed mitotic progression, including the induction of cell cycle alteration, retardation of cell abscission time, and regression of the cleavage furrow. In addition, we revealed that RRSP_Pa_ could target the cyclin-dependent kinase 1 (CDK1) protein and block activation of CDK1 through inhibition of Thr161 phosphorylation, which partially explained the crucial role of this effector in cell mitosis.

## Introduction

Contractile injection systems (CISs) are widely distributed in bacteriophages and bacteria, including the phage tail, the type VI secretion system (T6SS), the R-type pyocin, and others. The main function of CISs is to deliver genetic materials and effectors into both prokaryotic and eukaryotic hosts (Taylor et al., 2018). The structure, assembly, and mechanism of typical CISs, such as the contractile tail of bacteriophage T4, have been extensively investigated. This intracellular cell puncturing nanomachine consists of an inner tube capped by a spike, a contractile sheath, and a baseplate complex located at the base of the sheath (Leiman and Shneider, 2012). In addition, T6SSs are widespread in bacteria and can be used both for eukaryotic host cell attack and for interbacterial competition (Jiang et al., 2014, 2016). Most T6SSs are located in the cytoplasm, anchored to the inner membrane, and deliver toxin effectors to eukaryotic cells or bacteria (Jani and Cotter, 2010; Russell et al., 2011).

The “*Photorhabdus* virulence cassettes” (PVCs) produced by *Photorhabdus* spp. are distinct from intracellular CISs, which can be directly secreted into the medium and confer insecticidal activity against the wax moth (Yang et al., 2006). CISs with this type of action mode are defined as “extracellular CISs” (eCISs) (Nakayama et al., 2000; Ghequire and De Mot, 2015). The eCISs can be released outside of the bacteria to interact with the targeted cell surface. For example, the R-type pyocins secreted by *Pseudomonas aeruginosa* could function through pore formation in the envelope of competing bacterial cells (Nakayama et al., 2000; Michel-Briand and Baysse, 2002).

In previous work, we reported the cryogenic electron microscopy (cryo-EM) structure of an intact PVC particle from *Photorhabdus asymbiotica* ATCC43949 (Jiang et al., 2019). We demonstrated that the PVC device resembled a simplified T4 phage tail, comprising a hexagonal baseplate complex with six fibers and a capped 117-nm sheath-tube complex. A comparison of the structure and assembly process of PVC and other CISs indicated that PVC may be an evolutionary intermediate between the T4 phage and T6SS (Jiang et al., 2019). Compositional and structural analysis of PVCs has increased our understanding of eCISs. In the following work, we identified the PVC effectors and attempted to determine the function of the effectors in eukaryotic cells.

Multifunctional autoprocessing repeats-in-toxin (MARTX) toxins are effector delivery platforms playing critical roles in numerous Gram-negative bacteria. The MARTX toxins are composed of conserved repeat regions in N- and C-terminus and an autoprocessing protease domain to deliver the effector domains into eukaryotic cells (Egerer and Satchell, 2010; Gavin and Satchell, 2015; Satchell, 2015; Lee et al., 2019). Generally, one single MARTX toxin possesses a repertoire of up to five effector domains, organized in a characteristic modular fashion (Prochazkova et al., 2009; Shen et al., 2009). Several effector domains of MARTX have been characterized, such as the Rho GTPase-inactivation domain (RID) (Sheahan et al., 2004), actin cross-linking domain (ACD) (Fullner and Mekalanos, 2000), and Ras/Rap1-specific endopeptidase domain (RRSP, also known as DUF5) (Antic et al., 2014, 2015). The RRSP domain was identified in different *Vibrio vulnificus* isolates and other pathogens and specifically cleaved the switch I domain of the Ras and Rap1 proteins in eukaryotic cells (Antic et al., 2015).

In *P. asymbiotica*, we found a gene encoding a protein with homology to RRSP (RRSP_Pa_), which located downstream of the PVC cluster. The literature proved that the PVC can deliver the corresponding effectors in proximity to the locus (Yang et al., 2006; Vlisidou et al., 2019). This suggests that the RRSP_Pa_ could be potential PVC delivered effector. It is well established that the RRSP domain in MARTX toxin from *V. vulnificus* targets Ras for processing causing ERK1/2 dephosphorylation in HeLa cells (Antic et al., 2015). In this report, we showed that the RRSP_Pa_ protein was associated with inhibition of cell proliferation and increased cell apoptosis and death of HeLa cells. We demonstrated that RRSP_Pa_ induced cell cycle alteration, delayed cell mitotic progression, and led to regression of the cleavage furrow during cytokinesis. In addition, we found that RRSP_Pa_ targeted the cyclin-dependent kinase 1 (CDK1) protein and inhibited the CDK1 Thr161 phosphorylation in HeLa cells, which partially explained the crucial role of this effector domain in cell mitosis.

## Materials and Methods

### Bacterial Strains and Growth Conditions

The *P. asymbiotica* ATCC43949 strain was cultured in Luria–Bertani (LB) medium at 30°C. *Escherichia coli* strains were cultured in LB broth at 37°C unless indicated. The *E. coli* strains used in this study were DH5α for plasmid maintenance, BL21 (DE3) for immunoprecipitation (IP). Antibiotics were used as follows: 100 μg/ml ampicillin, 25 μg/ml chloramphenicol, and 25 μg/ml kanamycin.

### Antibody Reagents

Primary antibodies of mouse IgG1 monclonal anti-FLAG [Clone number: M2] (# ab49763), rabbit monoclonal anti-CDK1 antibody [Clone number: EPR165] (# ab133327), and rabbit monoclonal anti-CDK2 (phospho T160) + CDK1 (phospho T161) antibody [Clone number: EPR17621] (# ab183554) were purchased from Abcam. The primary rabbit monoclonal anti-GAPDH antibody [Clone number: D16H11] (# 8884) was purchased from Cell Signaling Technology (CST). Primary mouse monoclonal anti-Green Fluorescent Protein (GFP) antibody [Clone number: GSN149] (# G1546) was purchased from Sigma. The secondary horseradish peroxidase (HRP)-conjugated goat anti-rabbit IgG (heavy and light chain) antibody (# 7074) and the HRP-conjugated goat anti-mouse IgG (heavy and light chain) antibody (# 7076) were purchased from CST.

The Anti-DDDDK-tag mAb-Magnetic Agarose (M185-10) and anti-GFP magnetic beads Anti-GFP mAb-Magnetic Beads (D153-11) used for pull-down assay were ordered from Medical and Biological Laboratories (MLB).

### Plasmid Construction and Site-Directed Mutagenesis

The primers used to amplify the DNA fragment and site-directed mutagenesis are listed in Supplementary Table S1. The *RRSP*_Pa_ gene was amplified from the *P. asymbiotica* ATCC43949 genomic DNA as template (gene locus_tag:PAU_RS10135) with Q5 high-fidelity DNA polymerase (New England Biolabs). To construct the Flag-tagged RRSP_Pa_, Flag-tag was fused to the C terminus of *RRSP*_Pa_ gene in the PCR amplicons (primers of pcDNA3.1-F/R were used) and the *RRSP_Pa_-flag* were ligated into the pcDNA3.1 plasmid. For construction of *egfp* fusion plasmids, the PCR amplicons (primers of pEGFP-F/R were used) were cloned into pEGFP-C1 (Clontech) to generate in-frame fusions with *egfp*. For construction of plasmids expressing RRSP_Pa_ ΔMLD, DNA encoding the amino acids for RRSP_Pa_ 1–45 (primers of pEGFP-F/MLD-R were used) and 124–542 (primers of MLD-F/pEGFP-R were used) were separately amplified and fused together by overlap PCR to generate the DNA fragment encoding RRSP_Pa_ ΔMLD, and then the product was ligated into pEGFP-C1 for *egfp* fusion. The site-directed mutagenesis kit (Stratagene) was used to generate single amino acid codon substitutions. All mutations were verified by sequencing.

### Cell Culture and Transfection

HeLa and 293T cells were cultured in Dulbecco’s modified Eagle’s medium (DMEM, Thermo Scientific) supplemented with 10% fetal bovine serum (FBS). Cells were incubated in a humidified atmosphere with 5% CO_2_ at 37°C. Cell cultures were grown to indicated confluence, and subjected to transient transfection with Lipofectamine 3000 (Invitrogen) according to the manufacturer’s instructions. For analysis of proliferation and the cell cycle profile, the pcDNA3.1, pcDNA3.1-RRSP_Pa_, and pcDNA3.1-RRSP_Pa_ H485A plasmids were transfected into HeLa cells. For confocal microscopy, HeLa cells were transfected with various *egfp* fusion plasmids. For the immunoblotting assay, pEGFP and pEGFP-RRSP_Pa_ plasmids were transfected into HeLa cells.

### Fixed Cell Microscopy

For confocal microscopy, HeLa cells were cultured in glass bottom dishes overnight. After 16 h of transfection, cells were fixed with 4% paraformaldehyde in PBS for 10 min and permeabilized with 0.25% Triton X-100 for 10 min at room temperature, followed by blocking with 1% bovine serum albumin. For membrane staining, the cells were incubated with CellMask^TM^ orange plasma stain (Life Technology), washed three times with PBS, and subsequently stained with DAPI (Sigma) for 10 min and mounted for microscopic observation. The fixed cell confocal images were acquired on a Leica TCS SP5 confocal microscope using a 63 × (1.4 NA) oil immersion objective. The 488 nm laser and 510/20 nm bandpass (BP) emission filter were used for detection of EGFP; the 360 nm laser and 460/50 nm BP emission filter were used for detection of nuclei (DAPI). Image data were first analyzed with Leica Application Suite X (LAS X) software, for quantification of rounded cells, 300 cells were manually counted from three independent experiments (100 counted cells for each time) as described (Antic et al., 2014). The rounded cells were picked by using Image J software with the circularity between 0.6 and 1.0. Results were recorded and graphed as histograms with GraphPad Prism 7.0 software, and the image contrast was modified with Image J for better quality.

### Live Cell Microscopy

HeLa cells were cultured in glass bottom dishes overnight and then subjected to transfection with *egfp* fusion plasmids for 16 h, followed by live cell imaging. For phase contrast microscopy images, time-lapse live cell imaging was performed on a Nikon Ti2-Eclipse inverted microscope with a 10 × (0.45 NA) objective lens. For confocal microscopy images, nuclei were stained with Hoechst 33342 for 10 min and then mounted for microscopic observation with a Leica TCS SP5 microscope with a 63 × (1.4 NA) oil immersion objective. The microscopes were equipped with a chamber running at 37°C and delivering 5% CO_2_ for live cell imaging. The 488 nm laser and 510/20 nm BP emission filter were used for detection of EGFP; the 350 nm laser and 460/50 nm BP emission filter were used for detection of nuclei (Hoechst 33342). Both the phase contrast microscopy images and confocal images were captured every 10 min, subsequently analyzed with NIS-Element AR software and LAS X software, respectively. For quantification of cell duration and furrow regression, individual cells were tracked for 6 h and at least 50 cells that underwent mitosis were measured for each group as described (Moulding et al., 2007; Lim et al., 2013; Yuan et al., 2019). Cells observed that progressed to furrow ingression but subsequently regressed, leading to mitosis failure were counted as furrow regression cells. In our experiments, the cleavage furrow was defined visually when the cells entered the telophase and two daughter cells started to separate during the mitosis (Herrmann et al., 2003; Normand and King, 2010). Results were recorded manually and graphed as histograms with GraphPad Prism 7.0 software, and the image contrast was modified with Image J software for better quality.

### Flow Cytometry Analysis of Cell Cycle

The flow cytometry analysis was studied through the PI/RNase Staining Solution. HeLa cells were seeded in six-well culture plates overnight, transfected with pEGFP, pEGFP-RRSP_Pa_, and pEGFP-RRSP_Pa_ H485A, and then subjected to flow cytometry for cell cycle analysis. Cells were trypsinized with 0.25% trypsin (Hyclone), harvested by centrifugation, washed with cold PBS, and the cells were first fixed in 4% paraformaldehyde for 20 min at 4°C, following by fixation in 70% ice-cold ethanol. After fixation, cells were resuspended in PBS at a density of 1 × 10^6^ cells/ml and incubated with DNase-free RNase A (Sigma) at 37°C for 1 h. Following RNase digestion, cells were stained with propidium iodide (PI, Sigma) for 30 min in the dark. The software used an algorithm which attempt to fit Gaussian curves to each phase. Then each cell population showed a specific pattern in the PI histogram to differentiate the cell cycle phase: cells in a diploid (2N) state were defined as G1 phase, cells in a tetraploid (4N) state were defined as G2/M phase, and cells between these states (2N–4N) were defined as S state.

The BD LSRII instrument (BD Biosciences, San Jose, CA, United States) was used for flow cytometry analysis. Samples were excited with 488 nm laser, then a 505 nm long pass (LP) filter and a 530/30 nm BP filter were used to detect EGFP; a 550 nm LP filter and a 575/26 nm BP filter were used to detect PI. At least 20,000 events were counted for each experiment. Events were viewed on forward-scatter (FSC) versus side-scatter (SSC) plots with gating the single cell population, and then the gated events were viewed on fluorescein isothiocynate (FITC) versus SSC to collect the EGFP positive cells. Doublet events were eliminated from gating on phycoerythrin width (PE-W)/phycoerythrin area (PE-A) signals through the red filter before histogram analysis of DNA content. The NovoExpress software was used for cell cycle analysis of the EGFP positive cells.

### Flow Cytometry Analysis of Cell Apoptosis

For cell apoptosis analysis, HeLa cells were grown overnight, transfected, and harvested at indicated time. The harvested cells were washed twice with cold PBS and resuspended in 1 × Binding Buffer at a concentration of 1 × 10^6^ cells/ml and then 100 μl of the solution (about 1 × 10^5^ cells) were transferred to 5 ml tube. After that, 5 μl of FITC Annexin V and 5 μl PI were added into the tube and incubated for 15 min at room temperature in dark. Then 400 μl of 1 × Binding Buffer was added and the samples were analyzed by flow cytometry within 1 h. The reagents used for cell apoptosis analysis were all from BD Pharmingen^TM^ FITC Annexin V Apoptosis Detection Kit I (BD Biosciences). Samples were collected at different time points and the FACS gating was set according to different cell population and controls. Three groups of controls (cell without staining, cells stained with PI, cells stained with Annexin V) were used to set the cross hair and adjust the compensation.

### MTS Assay

HeLa cells were seeded in 96-well plates (seeding densities were 0.4 × 10^4^, 0.8 × 10^4^, and 1.6 × 10^4^ cells/well, respectively), grown overnight, and transfected with pcDNA3.1, pcDNA3.1-RRSP_Pa_, and pcDNA3.1-RRSP_Pa_ H485A. The viability was measured at A490 nm with the CellTiter 96^®^ Aqueous One Solution cell proliferation assay (MTS, Promega) according to the instructions at the indicated times (0, 12, 24, 36, 48, 60, and 72 h) after transfection.

### Mass Spectrometric Analysis

HeLa cells were transfected with pEGFP and pEGFP-RRSP_Pa_ H485A plasmids. After 16 h of transfection, cells were washed with PBS and then lyzed with RIPA buffer (CST) supplemented with protease and phosphatase inhibitor mixture (Roche) for 30 min. Then, cell lysates were incubated with anti-GFP magnetic beads (MBL) at room temperature for 2 h. After incubation, the beads were washed three times with PBST buffer (10 mmol/l phosphate buffer, 137 mmol/l NaCl, 2.7 mmol/l KCl, 0.05% Tween-20) and finally boiled in SDS-PAGE sample buffer to release the bound protein. Protein was separated via SDS-PAGE gels and stained with a silver staining kit (Invitrogen). The bands were excised and subjected to Nano LC-MS/MS analysis.

The instruments and parameters for nano LC were used as following: Nanoflow UPLC: Easy-nLC1000 (ThermoFisher Scientific, United States); Nanocolumn:100 μm × 15 cm in-house made column packed with a reversed-phase ReproSil-Pur C18-AQ resin (3 μm, 120 Å, Dr. Maisch GmbH, Germany); loaded sample volume: 5 μl; mobile phase: A: 0.1% formic acid in water; B: 0.1% formic acid in acetonitrile; total flow rate: 300 nl/min; LC linear gradient: from 6 to 9% B for 25 min, from 9 to 14% B for 60 min, from 14 to 30% B for 90 min, from 30 to 40% B for 60 min, and from 40 to 95% B for 15 min, eluting with 95% B for 10 min.

The instruments and parameters for mass spectrometry were used as following: Q Exactive^TM^ Hybrid Quadrupole-Orbitrap^TM^ Mass Spectrometer (Thermo Fisher Scientific, United States); spray voltage: 2.2 kV; capillary temperature: 270°C; MS parameters: MS resolution: 60,000 at 400 *m/z*; MS precursor *m/z*; range: 300.0–1650.0; MS/MS parameters: product ion scan range: start from *m/z* 100; activation type: HCD; min. signal required: 1500.0; isolation width: 3.00; normalized coll. energy: 40.0; default charge state: 6; activation Q: 0.250; activation time: 30.000; data-dependent MS/MS: up to top five most intense peptide ions from the preview scan in the Orbitrap.

### CDK1 Immunoblotting and Pull-Down Assay

For CDK1 immunoblotting, HeLa cells transfected with pEGFP and pEGFP-RRSP_Pa_, pEGFP-RRSP_P__a_ H485A plasmids were lyzed and incubated with anti-GFP magnetic beads as mentioned above. Then, the eluents were subjected to Western blot probing with primary antibodies against CDK1 and EGFP.

For the *in vivo* CDK1-RRSP_Pa_ pull-down assay, HeLa cells were cotransfected with EGFP*-*fused RRSP_Pa_ variants and Flag-tagged CDK1 (Origene). After transfection for 16 h, cells were lyzed and incubated with anti-FLAG magnetic beads, following by subjected to Western blot probing with primary antibodies against Flag and EGFP.

For the detection of the CDK1 phosphorylation, the primary antibodies were used as follows: anti-CDK1, anti-CDK1 (phospho T161), and anti-GAPDH. Assays were performed with three independent experiments.

## Results

### RRSP_Pa_ Inhibits Cell Proliferation

**FIGURE 1:**
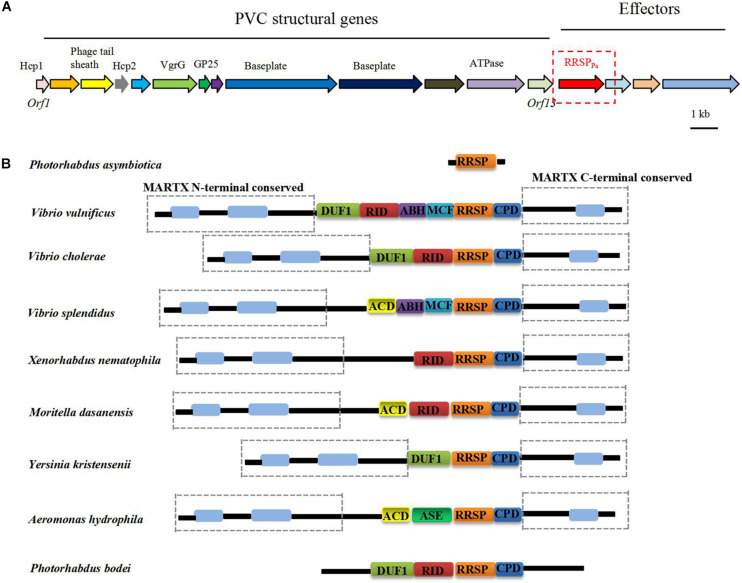
**(A)** Genomic organization of the PVC cluster and the location of the *RRSP*_Pa_ gene. **(B)** Distribution of RRSP_Pa_ homologs in toxins from representative pathogens. DUF1, domain of unknown function in the first position; RID, rho inactivation domain; ABH, alpha/beta hydrolase effector domain; MCF, makes caterpillars floppy-like; RRSP, Ras/Rap1-specific peptidase; CPD, cysteine protease domain; ACD, actin cross-linking domain; ASE, acetyl esterase/lipase.

Multiple PVC clusters were identified in the *P. asymbiotica* genome, and the PVC locus located upstream of the *RRSP*_Pa_ gene shown in Figure 1A. Figure 1B demonstrates that the homologous RRSP domains are widely distributed in the MARTX proteins from various pathogens (Antic et al., 2015). The membrane localization domain (MLD) of the four helical bundle family (4HB) (Geissler et al., 2010, 2012; Kamitani et al., 2010) was also identified within RRSP_Pa_ (Supplementary Figure S1).

The crystal structure of the MARTX toxin RRSP effector domain identified the conserved catalytic residues essential for enzymatic activity and cytotoxicity (Biancucci et al., 2018). Multiple amino acid sequence alignment of RRSP homologs with RRSP_Pa_ revealed that the catalytic residues were conserved across the proteins, and residues Glu-385 (E385) and His-485 (H485) were predicted to be the putative catalytic residues for RRSP_pa_ (Supplementary Figure S1).

Since RRSP_Pa_ shows homology to the RRSP domain, and the RRSP from *V. vulnificus* has been shown to induce cell rounding (Antic et al., 2014). We initiated our study by determining whether this domain induce eukaryotic cell rounding similarly to other RRSP homologs. The genes encoding RRSP_Pa_ and mutation variants (E385A, H485A, and ΔMLD, deletion of the 24–123 aa) (Kitadokoro et al., 2007) were cloned into a eukaryotic expression vector fused to *egfp* and then transiently expressed in HeLa cells. The results from three independent counting of rounding cell (Supplementary Figures S2A,B) and Western blots showing the transfection efficiency (Supplementary Figure S2C) were demonstrated. It was shown that 90% of cells transfected with EGFP-RRSP_Pa_ underwent robust cell rounding. In comparison, less cell rounding was detected when transfected with the E385A (40% rounded cells) and H485A (10.3% rounded cells) (Supplementary Figures S2A,B). Besides, cells producing EGFP-RRSP_Pa_ ΔMLD showed universal expression of RRSP within the entire cytoplasm instead of just at the plasma membrane. It was shown that only 21% of cells were rounded up when MLD domain was deleted (Supplementary Figures S2A,B), demonstrating that the protein localization could also affect the function of RRSP_Pa_.

**FIGURE 2:**
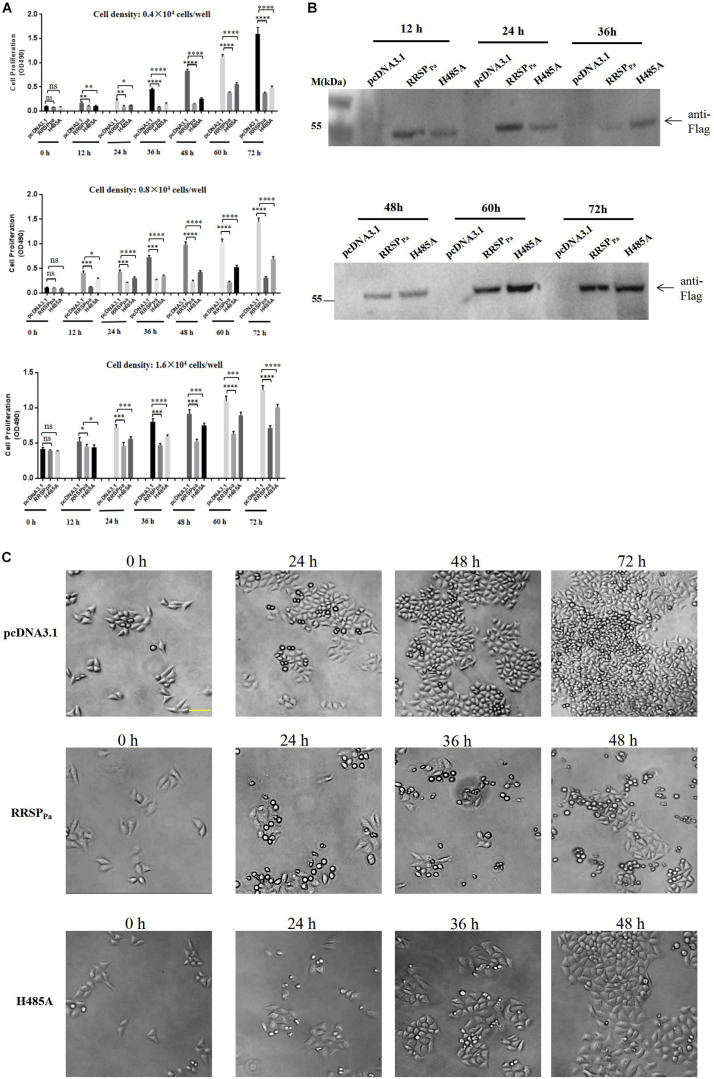
RRSP_Pa_ inhibits cell proliferation. HeLa cells were transfected with RRSP_Pa_ and H485A, and the pcDNA3.1 plasmid was transfected as a negative control. **(A)** The effect of RRSP_Pa_ and H485A on cell proliferation was measured by MTS assays at different time (0, 12, 24, 36, 48, 60, and 72 h) after transfection. **(B)** The expression levels of the RRSP_Pa_ and H485A mutant at indicated time were detected through Western blot with Flag antibody (cells seeding density: 0.8 × 10^4^ cells/well). **(C)** Light microscopic pictures (20 × magnification) were taken at the indicated time points (0, 24, 48, and 72 h) to demonstrate the confluence of cells at indicated time after transfection. Bar, 50 μm. The experiments were repeated three times, and *t*-test was conducted for statistical analysis, *P < 0.05; **P < 0.01; ***P < 0.001; ****P < 0.0001; ns, not significant.

To further determine the effect of RRSP_Pa_ on cell proliferation, different seeding density of HeLa cells were transfected with plamids of pcDNA3.1, pcDNA3.1-RRSP_Pa_ (RRSP_Pa_), pcDNA3.1-RRSP_Pa_ H485A (H485A), and MTS assays were performed to measure cell proliferation at different time points (0, 12, 24, 36, 48, 60, and 72 h) after transfection (Figure 2A). Western blots were performed to detect the expression level of the RRSP_Pa_ and H485A mutant at indicated time (Figure 2B). Moreover, cell growth was also monitored by light microscopy to show the effect of the RRSP_Pa_ on cell proliferation (Figure 2C). The initial cell densities used in the experiments were 0.4 × 10^4^, 0.8 × 10^4^, and 1.6 × 10^4^ cells/well, respectively. The results from three independent experiments are shown in Figure 2A demonstrating a significant inhibitory effect of RRSP_Pa_ on cellular proliferation over time. It was also observed that cells transfected with H485A mutant exhibited the growth defects when compared with the negative control (pcDNA3.1). Phase contrast images supported the cell proliferation results (Figure 2C). Therefore, these results suggested that RRSP_Pa_ inhibited the proliferation of HeLa cells.

### RRSP_Pa_ Induces Alteration of Cell Cycle Distribution and Promotes Apoptosis and Cell Death

**FIGURE 3:**
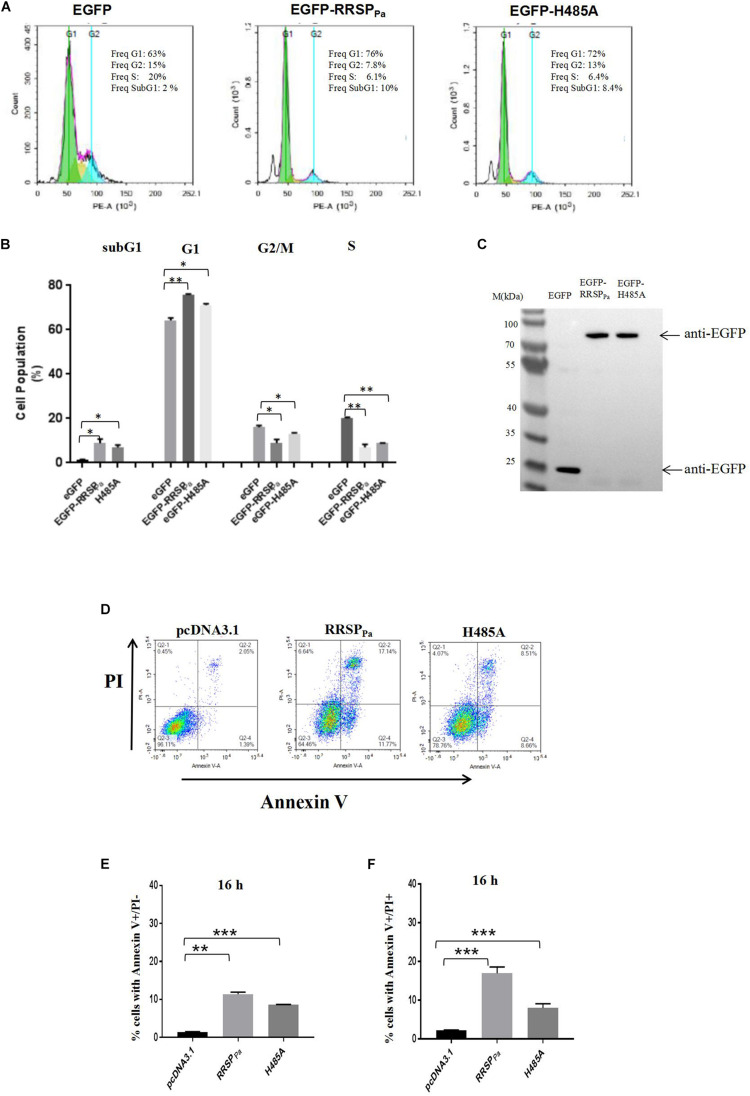
RRSP_Pa_ alters cell cycle distribution and increases apoptosis and cell death. **(A)** Representative cell cycle images of HeLa cells after transfection. HeLa cells transfected with pEGFP-RRSP_Pa,_ pEGFP-H485A, and pEGFP was used as a negative control. The cell cycle was analyzed by flow cytometry 16 h post-transfection. **(B)** The average percentage of cells in the cell cycle phases summarized from three biological replicates. **(C)** The expression levels of the EGFP, EGFP-RRSP_Pa_, and EGFP-H485A were detected through Western blot with primary GFP antibody. **(D)** Flow cytometric analysis of cells stained with Annexin V and PI. As identified by flow cytometry, cells were divided into four sections: upper left quadrant: Annexin V-/PI +, representing the mechanical error; upper right quadrant: Annexin V + /PI +, representing the late apoptosis or necrosis cells; lower left: Annexin V-/ PI-, representing the living cells; lower right quadrant: Annexin V + / PI-, representing the early apoptosis cells. **(E)** The percentage of cells with Annexin V + /PI-staining at 16 h. **(F)** The percentage of cells with Annexin V + /PI + staining 16 h. The data are presented as the mean and standard deviation from three independent experiments, and t-test was conducted for statistical analysis. *P < 0.05; **P < 0.01; ***P < 0.001.

To determine the mechanism underlying the RRSP_Pa_-mediated suppression of HeLa cell proliferation, we performed cell cycle distribution analysis through fluorescence-activated cell sorting (FACS) after 16 h of transfection. HeLa cells were transfected with pEGFP, pEGFP-RRSP_Pa_, or pEGFP-H485A, and only the GFP positive cells were gated for cycle analysis. Representative histograms demonstrating the cell cycle profile after transfection are shown in Figure 3A, and the 2D FACS dot plots and gating of EGFP positive cells for each histogram are shown in Supplementary Figure S3A. Cells treated with transfection reagent were used as internal control (Supplementary Figure S3B). Quantification of cell percentages in various phases of the cell cycle (Figure 3B) indicated that RRSP_Pa_ led to an obvious cell accumulation at the G1 stage, with an increase from 64 ± 0.8% for EGFP to 75 ± 0.3% for EGFP-RRSP_Pa_ transfected cells. Meanwhile, the H485A mutant-transfected cells showed 71 ± 0.5% of G1 stage cells (Figure 3B). Besides, there was a concomitant decrease in the percentage of cells in the S phase, as shown in Figure 3B, 20 ± 0.4% of control cells were in the S phase, while only 7.0 ± 0.9% of EGFP-RRSP_Pa_ and 8.8 ± 0.1% of EGFP-H485A transfected cells were at S stage. Western blots were also performed to detect protein expression of the EGFP, EGFP-RRSP_Pa_, and EGFP-H485A in cells used for cell cycle analysis (Figure 3C).

Moreover, it was observed that the percentage of cells at sub-G1-phase was increased in the pEGFP-RRSP_Pa_ transfected cells (8.8 ± 1.3%) as compared to the pEGFP (1.2 ± 0.2%) and pEGFP-H485A (6.9 ± 0.8%) transfected cells (Figures 3A,B). The sub-G1 fractions represent cells with fragmented DNA (cells in a hypodiploidy state), which is defined as an indication for cell apoptosis (Kondo et al., 2002; Wlodkowic et al., 2012). Therefore, we reasoned that the decreased cell proliferation may be a consequence of increased cell apoptosis.

Subsequently, cellular apoptosis was examined at 16 h after transfection, as assessed by annexin-V/PI through flow cytometry. We reported here that both the percentage of cells with annexin-V + /PI-staining (lower right quadrant, representing early cell apoptosis) and cells with annexin-V + /PI + staining (upper right quadrant, representing the late apoptosis and necrosis cells) were increased by RRSP_Pa_ (Figure 3D). As shown in Figure 3E, the index of early apoptosis (cell with annexin-V + /PI-staining) for RRSP_Pa_-transfected cells (11 ± 0.4%) was higher than that of negative control cells (1.4 ± 0.1%) and H485A-transfected cells (7.1 ± 0.1%) at 16 h after transfection. The rates of RRSP_Pa_-transfected cells with annexin-V + /PI + staining (cell death), as shown in Figure 3F, were 17 ± 0.9% occurring at 16 h. These were higher than that of negative control (2.2 ± 0.1%) and H485A-transfected (7.2 ± 0.6%) cells at 16 h. Consequently, these results indicated that RRSP_Pa_ altered the cell cycle distribution and promoted apoptosis and cell death.

### RRSP_Pa_ Causes Nuclear Abnormalities: Micronucleation and Nuclear Dysmorphology

As a defining characteristic of eukaryotic cells, the nucleus is bound by a membranous envelope and can be partitioned into two daughter cells during cell division. Cytological changes in the nucleus (such as multinucleation, micronucleation, nucleus structure alteration, etc.) are related to genome instability and mitotic catastrophe (Martins et al., 2012; Chircop, 2014).

**FIGURE 4:**
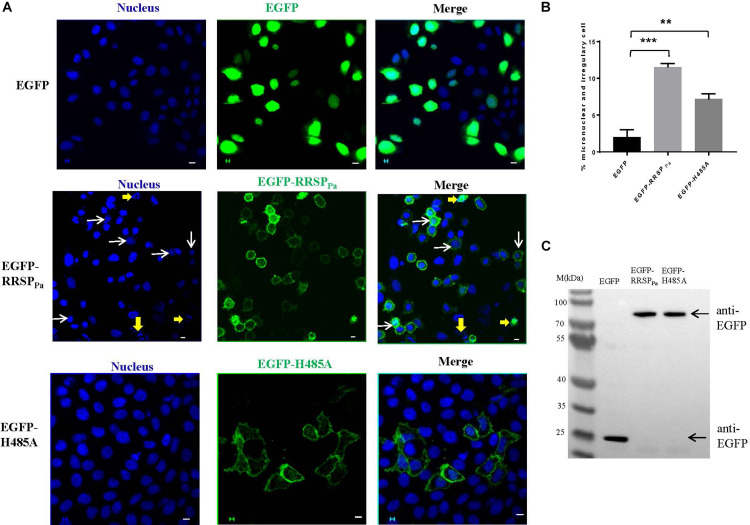
The cytogenetic abnormalities caused by RRSP_Pa_: micronucleation and irregularity of nuclear size and shape. HeLa cells were transfected with pEGFP-RRSP_Pa_ and pEGFP-H485A, and pEGFP was used as a negative control. **(A)** Visualization of micronucleation (yellow arrows) and irregularity of nuclear size and shape (white arrows) in the transfected cells. Bars, 5 μm. **(B)** Percentage of cells with micronucleation, and irregularity of nuclear size and shape. For each group, 300 cells were counted from three independent experiments (100 counted cells for each time). At least three individual images in each replicate were taken for counting. The data represent the mean and standard deviation of three experiments, and *t*-test was conducted for statistical analysis. **P < 0.01; ***P < 0.001. **(C)** The expression levels of the EGFP, EGFP-RRSP_Pa_, and EGFP-H485A were detected through Western blot with primary GFP antibody.

Here, HeLa cells were transfected with pEGFP, pEGFP-RRSP_Pa_, and pEGFP-H485A, respectively. Nuclear alteration was detected after 16 h of transfection. We found that the frequency of cells with nuclear defects was significantly increased by pEGFP-RRSP_Pa_ compared with that of cells expressing pEGFP and pEGFP-H485A. These defects included micronucleation and nuclei with an irregular size or shape. In the control cells transfected with EGFP, >97% displayed a stereotypic spherical nuclear morphology. Meanwhile, 11% of cells exhibited dysmorphology in the pEGFP-RRSP_Pa_-transfected cells, whereas the H485A mutant partially complemented this defect (7.2% defective cells) (Figures 4A,B). Western blots were performed to detect protein expression of the EGFP, EGFP-RRSP_Pa_, and EGFP-H485A in the transfected cells (Figure 4C).

Since the genomic stability is crucial for normal mitosis and cytokinesis, the findings of these nuclear abnormalities are suggestive of the substantial compromise of cell mitosis and cytokinesis.

### RRSP_Pa_ Delays Completion of Cell Mitosis and Causes Cell Death During Mitosis

**FIGURE 5:**
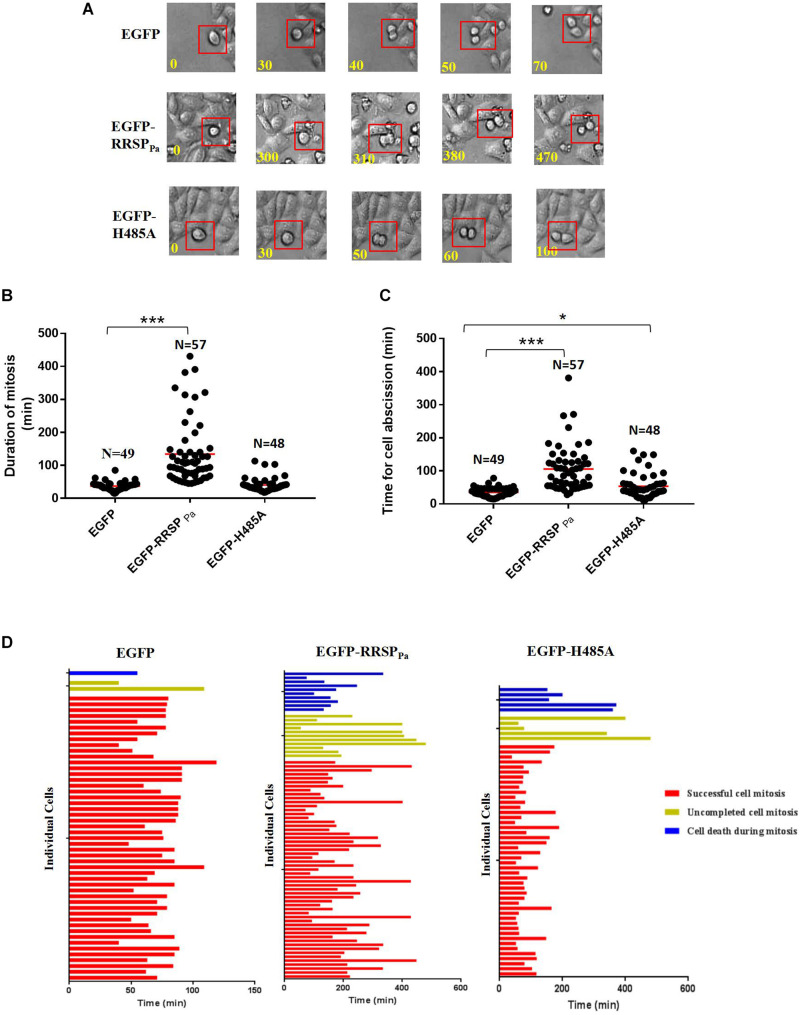
RRSP_Pa_ delays completion of cell mitosis and causes cell death during mitosis. HeLa cells were transfected with pEGFP-RRSP_Pa_, pEGFP-H485A, and pEGFP, respectively. 16 h after transfection, cells were monitored with phase-contrast, time-lapse live cell imaging for 8 h. **(A)** Representative frames from time-lapse series of HeLa cells expressing EGFP (top panel), EGFP-RRSP_Pa_ (middle panel), and EGFP-H485A (bottom panel); time was shown in minutes. The time from capturing the first image of a rounding up cell (*t* = 0 min) to the time of two daughter cells had visibly moved away from each another (ending of cell abscission) was measured. **(B)** Quantification of the duration of cell mitosis (from cell rounding up to anaphase onset). Time of each individual cell took to undergo mitosis was dotted, the solid red line represented the median time. **(C)** Quantification of the time for cell abscission (from anaphase onset to the time when the daughter cells separated, indicating the completion of abscission). Time of each individual cell took to undergo cell abscission was dotted, the solid red line represented the median time. **(D)** Fate profiles of cells entering mitosis that underwent successful mitosis, cell death, and incomplete cell mitosis. Each horizontal bar represented a single cell, and the color of the bar denoted the fate of the cell. The red bars indicated that the cells underwent successful cell mitosis, the yellow bar indicated uncompleted cell mitosis, and the blue bar indicated cell death during mitosis. The length of the bars denoted the duration of each cell fate. The length of red bar indicated the duration of time from cell rounding up to the completion of abscission. Time was shown in minutes. *N* representing the number of cells counted in this experiment. The experiments were repeated three times; at least three individual images in each replicate were taken for counting. *t*-test was used for statistical analysis. ***P < 0.001; *P < 0.05; ns, no significance.

To further investigate the effect of RRSP_Pa_ on cell cycle, live imaging was performed to score individual cells passing through mitosis. Representative frames from serial time-lapse imaging (Figure 5A) and quantitative analysis (Figures 5B,C) for cell division showed that cells transfected with pEGFP-RRSP_Pa_ spent a significantly longer period of time in mitosis and caused more cell death during mitosis when compared with EGFP and EGFP-H485A expressing cells.

We found that both the duration of mitosis (time elapsed from first image of a cell rounding up to anaphase onset) (Figure 5B) and cell abscission (from anaphase onset to the completion of cell abscission) (Figure 5C) were significantly extended by the RRSP_Pa_ production. Regarding the cells completing the mitosis, only one of 49 EGFP expressing cells and three of 46 EGFP-H485A expressing cells took longer than 60 min for mitosis duration, comparing with 48 out of 57 for EGFP-RRSP_Pa_ expressing cells. Average mitosis duration for EGFP and EGFP-H485A expressing cells were 37 ± 1.8 and 41 ± 3.1 min, respectively, whereas EGFP-RRSP_Pa_ cells experienced 134 ± 13 min (Figure 5B). Moreover, a dramatic delay was observed from the onset of telophase for cells expressing EGFP-RRSP_Pa_, indicating the dysregulation of the abscission. In 20 of 57 EGFP-RRSP_Pa_ cells, two rounded daughter cells stopped at the abscission stage and then experienced a retardation of longer than 120 min before separating. Average abscission time for EGFP and EGFP-H485A expressing cells were 35 ± 2.0 and 54 ± 5.4 min respectively, whereas cells expressing EGFP-RRSP_Pa_ took 106 ± 9 min for cell abscission (Figure 5C).

Cell fate profiles (Figure 5D) demonstrated different cell fate profiles in the EGFP-RRSP_Pa_ expressing cells comparing with EGFP and EGFP-H485A expressing cells. It was shown that one of 50 EGFP (death rate 2%) and five of 56 EGFP-H485A (death rate 9%) expressing cells entered mitosis and eventually death, while 11 of 78 EGFP-RRSP_Pa_ (death rate 14%) expressing cells underwent cell death.

### RRSP_Pa_ Leads to Regression of the Cleavage Furrow

As the final step in cell division, cytokinesis is the physical partition of the mother cell to generate two distinct daughter cells (Normand and King, 2010; Green et al., 2012). To explore how RRSP_Pa_ contributes to cytokinesis in HeLa cells, we followed the fate of pEGFP-RRSP_Pa_ transfected HeLa cells under phase-contrast time-lapse microscopy compared to that of control cells.

**FIGURE 6:**
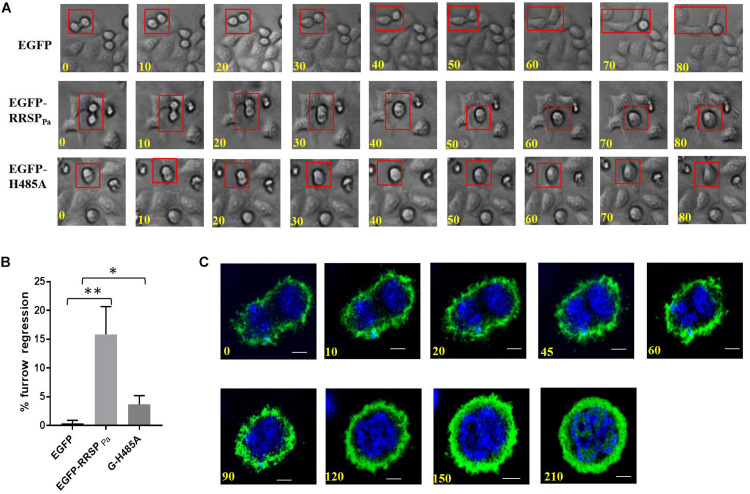
Furrow regression during cytokinesis induced by RRSP_Pa_. HeLa cells were transfected with pEGFP-RRSP_Pa_ and pEGFP-H485A, and pEGFP was used as a negative control. **(A)** Representative images from phase-contrast time-lapse recordings of HeLa cells producing EGFP (top panel), EGFP-RRSP_Pa_ (middle panel), and EGFP-H485A (bottom panel). **(B)** Percentage of observed live cells that exhibited cleavage furrow regression. The experiments were repeated three times and at least 50 cells were counted for each group, at least three individual images in each replicate were taken for counting. *t*-test was used for statistical analysis. *P < 0.05; **P < 0.01. **(C)** Confocal time-lapse imaging for the pEGFP-RRSP_Pa_-transfected cells showing the progress of the furrow regression.

The results showed that in all of the EGFP-expressing cells examined, the ingressed furrows were maintained until cytokinesis was completed, suggesting that EGFP did not affect cell cytokinesis. In contrast, in 16 ± 3% of cells transfected with EGFP-RRSP_Pa_, the furrows were formed and ingressed but subsequently regressed, leading to mitosis failure (Figures 6A,B). The regression of the cleavage furrow was also observed in 3.7 ± 1% of EGFP-H485A transfected cells. We confirmed the progress of cytokinesis with confocal time-lapse imaging (Figure 6C).

These results indicated that in HeLa cells, RRSP_Pa_ production caused regression of the cleavage furrow and disruption of cytokinesis progression at various time points.

### RRSP_Pa_ Interacts With CDK1 in Host Cells

To further explore the molecular mechanism of RRSP_Pa_-mediated mitotic defects in HeLa cells, we sought to identify the proteins that directly interacted with RRSP_Pa_ in HeLa cells through IP. Plasmids expressing *egfp* and *egfp-RRSP_Pa_* H485A were transfected into HeLa cells; then we analyzed the precipitated proteins by mass spectrometry to identify the putative RRSP_Pa_ binding proteins. The results showed that 13 proteins (e.g., ATP-dependent RNA helicase, DDX39A; exportin-2, CSE1L; vacuolar protein sorting-associated protein 51, VPS51; importin subunit beta-1, KPNB1; CDK1, etc.) were significantly abundant in the IP proteins of pEGFP-RRSP_Pa_ H485A transfected cells but not in those of the pEGFP transfected cells (fold change > 20) (Supplementary Table S2). Among these proteins, the CDK1 is regarded as a universal mitotic regulator (Nurse, 1990). We therefore chose it as a potential interaction partner of RRSP_Pa_ in the HeLa cells.

**FIGURE 7:**
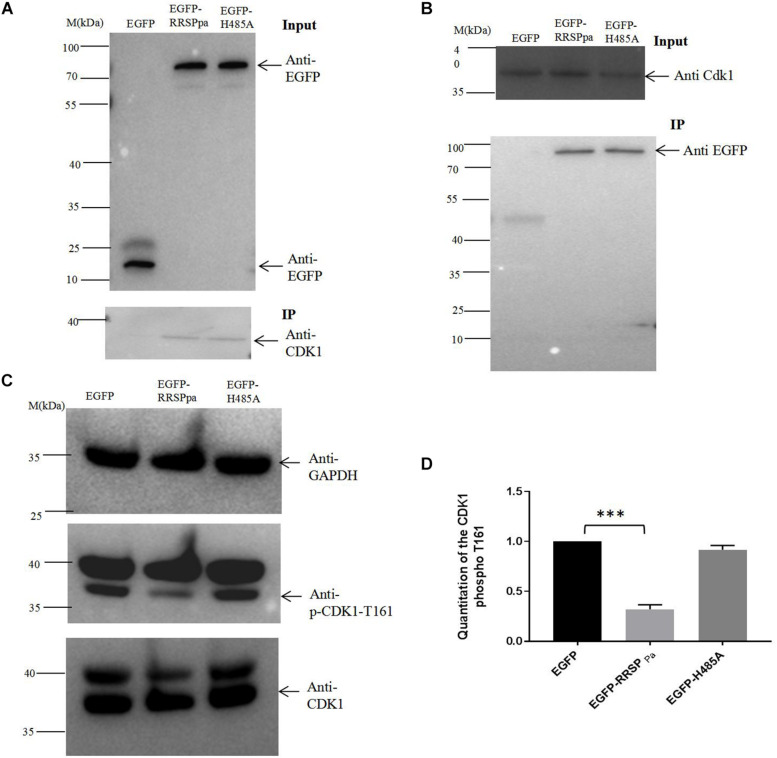
Interaction of RRSP_Pa_ with CDK1 by pull-down assays and the inhibition of CDK1 activity. **(A)** HeLa cells were transfected with pEGFP, pEGFP-RRSP_Pa_, and pEGFP-H485A. The top panel shows the expression levels of EGFP, EGFP-RRSP_Pa_, and EGFP-H485A in HeLa cells. The bottom panel shows the pull-down results. HeLa cell lysate was incubated with GFP-conjugated magnetic beads; immunoprecipitation was then conducted by blotting with anti-EGFP or anti-CDK1 antibodies. **(B)** HeLa cells were cotransfected with CDK1-Flag and EGFP, EGFP-RRSP_Pa_, or EGFP-H485A, respectively. The top panel shows the expression levels of CDK1. The bottom panel shows the pull-down results. HeLa cell lysates were incubated with Flag-conjugated magnetic beads; immunoprecipitation was conducted by blotting with anti-CDK1 or anti-GFP antibody. **(C)** HeLa cells were transfected with different plasmids, cell lysates were prepared and the levels of CDK1, CDK1 phospho T161, GAPDH were detected by Western blot analysis. The experiments were repeated three times and the representative pictures were shown. **(D)** Quantitation of the intensity of the CDK1 phospho T161 protein bands in **C**. *t*-test was used for statistical analysis. ***P < 0.001.

The coimmunoprecipitation analysis was performed to validate the protein–protein interaction between RRSP_Pa_ and CDK1. HeLa cells were transfected with plasmid of pEGFP, pEGFP-RRSP_Pa_, or pEGFP-H485A. IP of cell lysates incubating with GFP magnetic beads followed by western blot analysis showed that CDK1 protein coprecipitated with the EGFP-RRSP_Pa_ and EGFP-H485A proteins but not with the EGFP protein (Figure 7A). Conversely, RRSP_Pa_ was detected in the CDK1 IP samples. Cells were cotransfected with plasmids expressing Flag-tagged CDK1 and EGFP, EGFP-RRSP_Pa_, or EGFP-H485A. IP of cell lysates incubating with Flag agarose beads followed by western blot confirmed that the RRSP_Pa_ and CDK1 formed protein–protein association and this association was not disrupted by the H485A mutation (Figure 7B).

Coordinated regulation of CDK1 activity is crucial for mitotic progression (Timofeev et al., 2010). To detect the CDK1 activity, we conducted Western blots with CDK1 (phospho T161) antibody in crude lysates prepared from cells tranfected with plamids of pcDNA3.1, pcDNA3.1-RRSP_Pa_, or pcDNA3.1-RRSP_Pa_ H485A. Significant decreases of the phosphorylated Thr161 were observed in cells expressing RRSP_Pa_ comparing with the pcDNA3.1 and H485A transfected cells (Figures 7C,D). Since CDK1 Thr161 phosphorylation stabilizes interactions with downstream cyclins allowing for further activation (Ducommun et al., 1991), these data demonstrated that the RRSP_Pa_ overexpression blocked activation of CDK1 through inhibition of Thr161 phosphorylation.

## Discussion

A previous study has revealed that the RRSP domain could cleave the Ras and Rap1 proteins and subsequently inhibit the phosphorylation of ERK1/2 in eukaryotic cells (Antic et al., 2015). In this work, we discovered an alternative function of RRSP_Pa_ in eukaryotic cells. By observing the significant decrease of viable cell number in HeLa cells producing RRSP_Pa_, we speculated and proved that the RRSP_Pa_ was closely related to cell mitosis defects. These findings represent an additional aspect of RRSP_Pa_ function during the cell cycle.

Cell rounding was observed in cells expressing RRSP_Pa_ and homologs from other bacteria toxins (Antic et al., 2014), while the mechanism for this phenotype remained unclear. We found that cell rounding may partially due to the cell mitotic defects induced by RRSP_Pa_. During live imaging, it was observed that the “round stage” of the RRSP_Pa_ expressing cells was much longer than the control cells. When transfected with EGFP, cells rounded up and then entered to anaphase within 30 min whereas the EGFP-RRSP_Pa_ expressing cells experienced at least 2 h of the “round stage.” Moreover, commonly after abscission phase, the EGFP expressing cells returned to normal morphology in short time, whereas the RRSP_Pa_ expressing cells remained the rounding morphology for long time. Together, this may interpret the phenotype of cell rounding induced by RRSP_Pa_ (Figures 5A–C).

Besides the rounding morphology, the confocal microscopy images also demonstrated increased membrane blebbing in cells producing EGFP-RRSP_Pa_. Since the membrane blebbing indicates cell necrosis (Nagarajan et al., 2019), this observation was consistent with the cell death (late apoptosis and cell necrosis, Figures 3D–F) detected through flow cytometry.

CDK1 is a universal mitotic regulator (Nurse, 1990) and drives cell cycle progression when bound to its partner cyclin B (Murray, 1994; Mishima et al., 2004; Zhu and Jiang, 2005; Estey et al., 2013; Mathieu et al., 2013). In this work, we demonstrated that RRSP_Pa_ influenced the CDK1 Thr161 phosphorylation in HeLa cells. As documented previously, CDK1 was related to cytokinesis failure and cell apoptosis, which partially explained the crucial role of this effector for cell mitosis. Thus, the RRSP_Pa_ induced cell cycle alteration and apoptosis would ascribe to the CDK1 down-regulation by RRSP_Pa_. It was shown that the results of H485A mutation were significantly different from the negative control of pcDNA3.1 and pEGFP, besides the protein–protein interaction analysis showed that the H485A mutation did not affect the interaction with CDK1. These data demonstrated that RRSP_Pa_ association with CDK1 was independent of cleavage of Ras and Rap1, revealing an additional mechanism of action for the Ras/Rap1 protease.

It was reported that CDK1 controls proteins involved in DNA replication and chromosome segregation (Enserink and Kolodner, 2010). Actually, we have observed the chromosome abnormalities induced by RRSP_Pa_ through confocal imaging of cells in anaphase and telophase, such as the centrophilic chromosome, lagging chromosome, and misaligned chromosomes (data not shown). Since the chromosome abnormalities are related to cytokinesis failure and cell death during mitosis (Normand and King, 2010), the cell mitotic catastrophe may not only result from the inhibition of CDK1 Thr161 phosphorylation but also the chromosome defects due to premature activation of CDK1. Hence, the detailed molecular mechanism of RRSP_Pa_ induced mitotic catastrophe is comprehensive and remains to be further elucidated.

Furthermore, we observed that several putative effectors encoded by genes downstream of the PVC clusters were homologous to effectors of type III secretion systems (T3SSs). For example, the gene encoding the YopT homologous protein was located downstream of the same PVC cluster as *RRSP*_Pa_ (Yang et al., 2006). In pathogenic *Yersinia* species, the YopT effector protein can be delivered through a T3SS into host cells and functions as a cysteine protease to cleave Rho family GTPases (RhoA, Rac, and Cdc42) (Shao et al., 2003). Moreover, a gene encoding homologs of the T3SS effector GogB is also present in the *P. asymbiotica* genome. A recent finding suggest that a cytotoxic necrotizing factor (CNF) homologous protein, Pnf, could be delivered by the PVC needle complex to exert its effect (Vlisidou et al., 2019). Given this finding, we hypothesized that the PVC structure proteins and effectors evolved from different ancestors, as the structure of PVC is considered to be an evolutionary intermediate between the T4 phage and T6SS (Jiang et al., 2019), while the effectors may be horizontally transferred from other secretion systems, such as T3SSs or other systems.

## Data Availability Statement

The raw data supporting the conclusions of this article will be made available by the authors, without undue reservation, to any qualified researcher.

## Author Contributions

XW, FJ, and QJ conceived the study and designed experimental procedures. XW, JS, and FJ performed the experiments and carried out data analysis. XW and FJ wrote the manuscript.

## Conflict of Interest

The authors declare that the research was conducted in the absence of any commercial or financial relationships that could be construed as a potential conflict of interest.

## Supplementary Material

FIGURE S1 Multiple sequence alignment of RRSP_Pa_ homologs. The black boxes and asterisks indicated the residues critical for cell rounding (E385 and H485); the blue underline indicated the MLD domain. Residues are numbered according to the sequence of RRSP_Pa_, alignment was generated using Clustal Omega, alignment shading was done by ESPript, and the sequences with red background represented identical residues. The sequences used were all from NCBI with accession numbers as follows: NCBI # WP_015834234.1 in *Photorhabdus asymbiotica*; NCBI # WP_038941175.1 in *Vibrio vulnificus*; NCBI # WP_114967676.1 in *V. cholerae*; NCBI # WP_108151088.1 in *V. splendidus*; NCBI # WP_112896878.1 in *Photorhabdus bodei*; NCBI # WP_041979239.1 in *Xenorhabdus nematophila*; NCBI # WP_017223230.1 in *Moritella dasanensis*; NCBI # WP_004390551.1 in *Yersinia kristensenii*; NCBI # WP_099369028.1 in *Aeromonas hydrophila*; NCBI # WP_003404659.1 in *Pseudomonas syringae*.

FIGURE S2 Ectopic expression of RRSP_Pa_ induces cell rounding, and E385 and H485 are critical for the induction. **(A)** Morphological changes of HeLa cells induced by the expression of EGFP, EGFP-RRSP_Pa_, EGFP-ΔMLD, EGFP-E385A, and EGFP-H485A. Bars, 5 μM. **(B)** Percentage of cells with rounded morphology. For each group, 300 cells were counted from three independent experiments (100 counted cells for each time). The data represent the mean and standard deviation of three experiments, and one-way ANOVA was used for statistical analysis. ****P* < 0.001. **(C)** Western blot using anti-GFP antibody with transfected cell lysates (from left to right: marker, EGFP, EGFP-RRSPPa, EGFP-ΔMLD, EGFP-E385A, and EGFP-H485A).

FIGURE S3 **(A)** The 2D FACS dot plots and gating of EGFP positive cells for each histogram. The FSC-A versus SSC-A plot were used to gate the single cells population (upper left graph), subsequently EGFP positive cells were gated thorough FITC-A versus SSC-A criteria (upper right graph), doublet events were eliminated from gating on PE-W/PE-A primary plots (lower left graph) before histogram analysis of DNA content. Abbreviations: FSC-A, forward scatter area; SSC-A, side scatter pulse area; FITC-A, fluorescein isothiocynate area; PE-W, phycoerythrin width; PE-A, phycoerythrin area. **(B)** Representative histogram for the internal control. Cells treated with transfection reagent (Lipo3000) were used as an internal control.

TABLE S1 The primers used to amplify the DNA fragment and site-directed mutagenesis.

TABLE S2 MS analysis of proteins coimmunoprecipitated with EGFP and EGFP-RRSP_Pa_ H485A in the HeLa cells.
