# Corrigendum: The *Photorhabdus* virulence cassettes RRSP-like effector interacts with cyclin-dependent kinase 1 and causes mitotic defects in mammalian cells

**DOI:** 10.3389/fmicb.2023.1302833

**Published:** 2023-10-10

**Authors:** Xia Wang, Jiawei Shen, Feng Jiang, Qi Jin

**Affiliations:** NHC Key Laboratory of Systems Biology of Pathogens, Institute of Pathogen Biology, Chinese Academy of Medical Sciences and Peking Union Medical College, Beijing, China

**Keywords:** *Photorhabdus asymbiotica*, PVC, effector, RRSP, cell mitosis

In the published article, there were errors in Supplementary Figure S2. In Figure S2A, the incorrect picture was provided for EGFP-E385A. In Figure S2C, the annotation for the lane of the EGFP-H485A sample was missing. The correct supplementary material is published alongside the original article.

The authors apologize for this error and state that this does not change the scientific conclusions of the article in any way. The original article has been updated.

