# Corrigendum: The *Photorhabdus* virulence cassettes RRSP-like effector interacts with cyclin-dependent kinase 1 and causes mitotic defects in mammalian cells

**DOI:** 10.3389/fmicb.2024.1365940

**Published:** 2024-01-16

**Authors:** Xia Wang, Jiawei Shen, Feng Jiang, Qi Jin

**Affiliations:** NHC Key Laboratory of Systems Biology of Pathogens, Institute of Pathogen Biology, Chinese Academy of Medical Sciences and Peking Union Medical College, Beijing, China

**Keywords:** *Photorhabdus asymbiotica*, PVC, effector, RRSP, CELL mitosis

In the published article, acknowledgments were omitted in error. The correct Acknowledgments statement appears below.

## Acknowledgments

We thank Guowei Yang and Nicholas R. Waterfield for the *P. asymbiotica* ATCC43949 strain and helpful communications.

The authors apologize for this error and state that it does not change the scientific conclusions of the article in any way. The original article has been updated.

