# Supplementary figures and images for: The Photorhabdus Virulence Cassettes RRSP-Like Effector Interacts With Cyclin-Dependent Kinase 1 and Causes Mitotic Defects in Mammalian Cells
Source: Front Microbiol. 2020 Mar 13;11:366. doi: 10.3389/fmicb.2020.00366 (PMC7082817; doi:10.3389/fmicb.2020.00366)

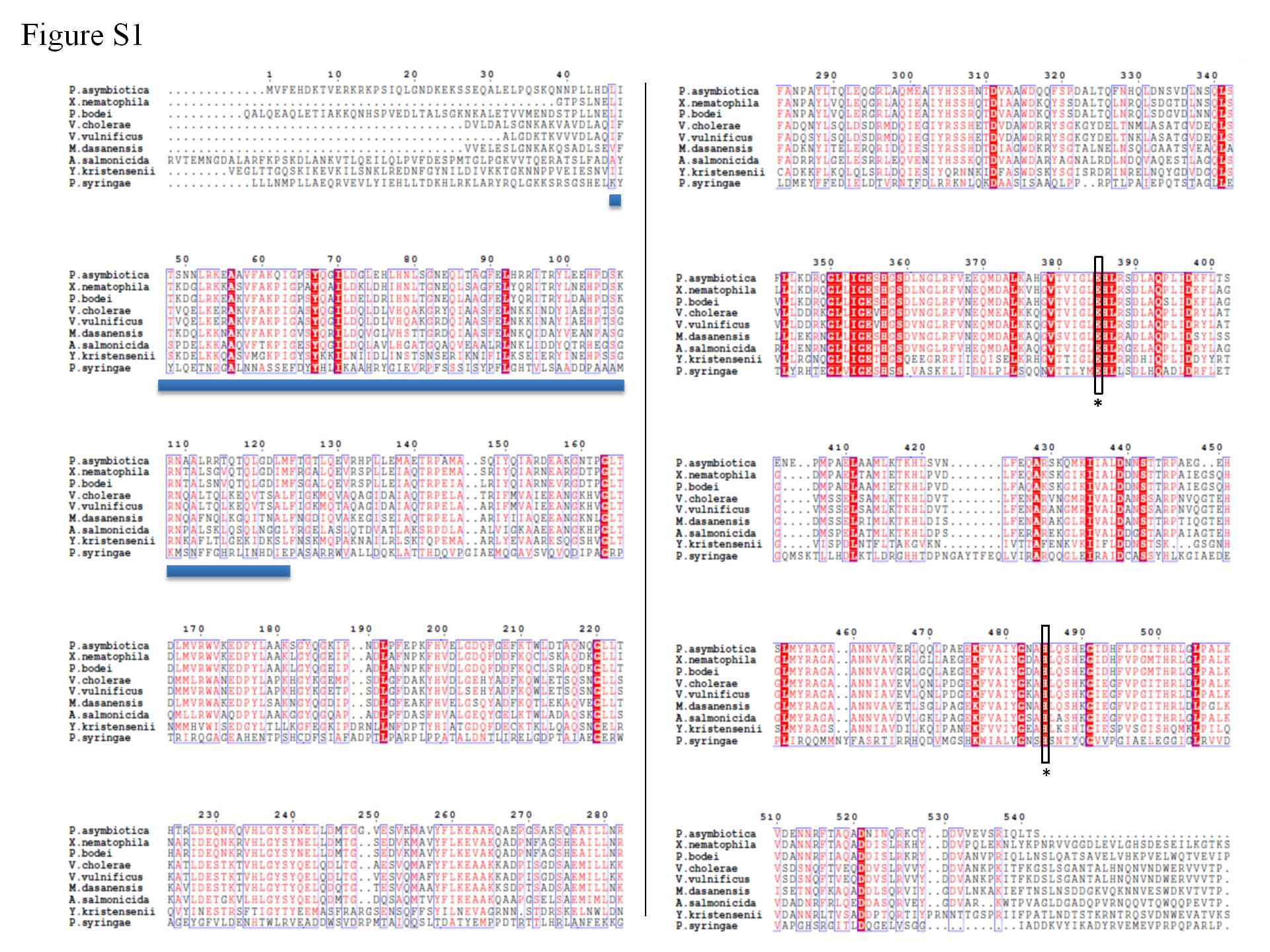

Supplement: FIGURE S1 — Multiple sequence alignment of RRSPPa homologs. The black boxes and asterisks indicated the residues critical for cell rounding (E385 and H485); the blue underline indicated the MLD domain. Residues are numbered according to the sequence of RRSPPa, alignment was generated using Clustal Omega, alignment shading was done by ESPript, and the sequences with red background represented identical residues. The sequences used were all from NCBI with accession numbers as follows: NCBI # WP_015834234.1 in Photorhabdus asymbiotica; NCBI # WP_038941175.1 in Vibrio vulnificus; NCBI # WP_114967676.1 in V. cholerae; NCBI # WP_108151088.1 in V. splendidus; NCBI # WP_112896878.1 in Photorhabdus bodei; NCBI # WP_041979239.1 in Xenorhabdus nematophila; NCBI # WP_017223230.1 in Moritella dasanensis; NCBI # WP_004390551.1 in Yersinia kristensenii; NCBI # WP_099369028.1 in Aeromonas hydrophila; NCBI # WP_003404659.1 in Pseudomonas syringae. [file Image_1.JPEG]

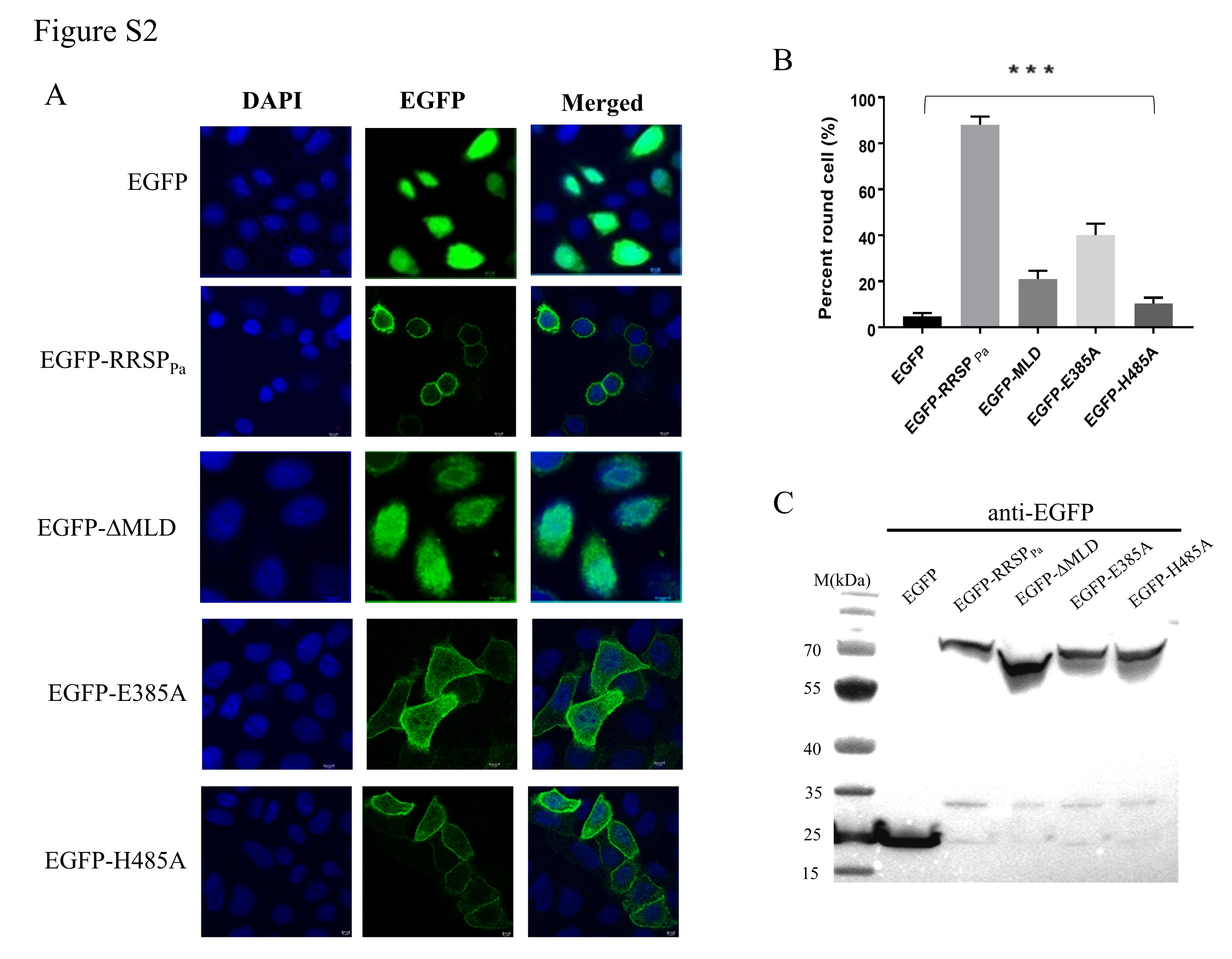

Supplement: FIGURE S2 — Ectopic expression of RRSPPa induces cell rounding, and E385 and H485 are critical for the induction. (A) Morphological changes of HeLa cells induced by the expression of EGFP, EGFP-RRSPPa, EGFP-ΔMLD, EGFP-E385A, and EGFP-H485A. Bars, 5 μM. (B) Percentage of cells with rounded morphology. For each group, 300 cells were counted from three independent experiments (100 counted cells for each time). The data represent the mean and standard deviation of three experiments, and one-way ANOVA was used for statistical analysis. ***P < 0.001. (C) Western blot using anti-GFP antibody with transfected cell lysates (from left to right: marker, EGFP, EGFP-RRSPPa, EGFP-ΔMLD, EGFP-E385A, and EGFP-H485A). [file Image_2.jpeg]

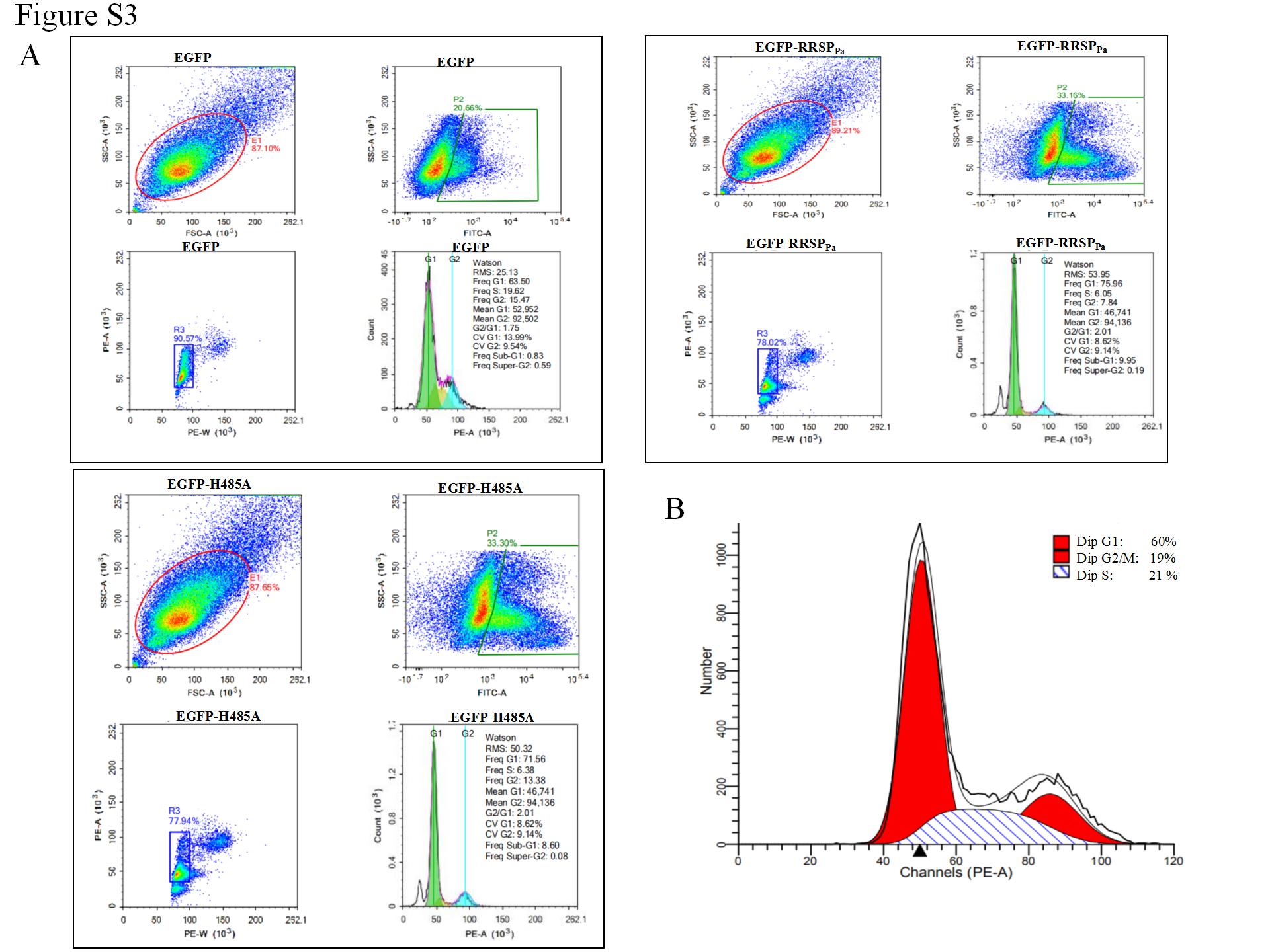

Supplement: FIGURE S3 — (A) The 2D FACS dot plots and gating of EGFP positive cells for each histogram. The FSC-A versus SSC-A plot were used to gate the single cells population (upper left graph), subsequently EGFP positive cells were gated thorough FITC-A versus SSC-A criteria (upper right graph), doublet events were eliminated from gating on PE-W/PE-A primary plots (lower left graph) before histogram analysis of DNA content. Abbreviations: FSC-A, forward scatter area; SSC-A, side scatter pulse area; FITC-A, fluorescein isothiocynate area; PE-W, phycoerythrin width; PE-A, phycoerythrin area. (B) Representative histogram for the internal control. Cells treated with transfection reagent (Lipo3000) were used as an internal control. [file Image_3.JPEG]
