# Supplementary material for: The Photorhabdus Virulence Cassettes RRSP-Like Effector Interacts With Cyclin-Dependent Kinase 1 and Causes Mitotic Defects in Mammalian Cells
Source: Front Microbiol. 2020 Mar 13;11:366. doi: 10.3389/fmicb.2020.00366 (PMC7082817; doi:10.3389/fmicb.2020.00366)
Supplement: TABLE S1 — The primers used to amplify the DNA fragment and site-directed mutagenesis. [file Table_1.pdf]

Table S1

Name	Primer
pcDNA3.1-F	GCCGCGGCCGCCGCCACCATGGTATTTGAGCACGATAAAAC
pcDNA3.1-R	GCCGATATC CTTGTCATCA TCGTCCTTGT AGTCAGATGTTA ATTGAATACGGG
pEGFP-F	GCCGGATCCATGGTATTTGAGCACGATAAAAC
pEGFP-R	GCCGTCGACCTAAGATGTTA ATTGAATACG GG
E385A-F	CATGGCGTGACAGTCATTGGTC TGGCGCACTT ACGCTCAGA
E385A-R	GCCAGACCAATGACTGTCAC GCCATGCGCT TTTAATG
H485A-F	GTT TGTTGCCATTTATGGTAATG CTGCTCTGCA
H485A-R	GCAGCATTAC CATAAATGGC AACAAACTTC TCTTCC
MLD-L	CCATTACTCC ATGACCTCAT CACCAGCAAT GCCCTGAGAA GAACACAGAC
MLD-R	CCCCAATTGC GTCTGTGTTC TTCTCAGGGC ATTGCTGGTG ATGAGGTCAT
